# Rhymes of Science

**Published:** 1879-06

**Authors:** 


*Rhymes of Science:* Wise and Otherwise, with illustrations. Published by the "Industrial Publication Company," New York, 1879.

A small volume of Scientific Poetry, by O. W. Holmes, Bret Hart, Ingoldsby, Prof. Forbes, Prof. I. H. McQ. Rankine, Hon. R. W. Reynolds, and others. Price 50 cents.

